# Trust in nutrition, subjective norms and urban consumers’ purchase behavior of quinoa products: explanation based on preference heterogeneity

**DOI:** 10.3389/fnut.2024.1511205

**Published:** 2024-12-04

**Authors:** Chan Wang, Xiaoyan Fu, Caixia Li, Zengjin Liu, Shanshan Wang, Tinggui Chen, Lei Jia

**Affiliations:** ^1^Institute of Information of Agricultural Science and Technology, Shanghai Academy of Agricultural Sciences, Shanghai, China; ^2^Shanghai Key Laboratory of Agricultural Genetics and Breeding, Biotechnology Research Institute, Shanghai Academy of Agricultural Sciences, Shanghai, China; ^3^College of Economics and Management, Shanghai Ocean University, Shanghai, China

**Keywords:** trust in nutrition, subjective norms, quinoa, purchase behavior, moderating effect

## Abstract

**Introduction:**

With the increase of disposable income and the awakening of health consciousness of Chinese residents, higher requirements have been put forward for the nutritional value of food. To meet the market demand and promote the high-quality development of the whole quinoa industry chain, this study aimed to analyze the purchase behavior of quinoa from the aspects of nutritional trust and subjective norms.

**Methods:**

Based on 1,078 micro-survey data from 16 administrative districts in Shanghai, this paper empirically examined the influence of trust in nutrition and subjective norms on consumers’ purchase behavior and willingness to pay of quinoa products, and further investigated the moderating effect of preference heterogeneity.

**Results:**

Results show that 38.22% of respondents have purchased quinoa products. Trust in nutrition and subjective norms can not only promote their purchasing behavior, but also improve consumers’ willingness to pay for quinoa products. The analysis of the moderating effect shows that both the purchase behavior and the willingness to pay are affected by the preference heterogeneity, and the cognitive preference will weaken the positive influence of subjective norms on the purchase behavior, while the nutritional preference of consumers can strengthen the positive influence of trust in nutrition on the willingness to pay.

**Discussion:**

Therefore, this paper suggests that strengthens research and development of products market oriented, implements certification and labeling schemes for nutrition and other properties of products, intensifies publicity and promotion of products to enhancing consumers’ awareness of healthy diet.

## 1 Introduction

Quinoa is quite comprehensive regarding nutritional content, and its taste texture and flavor are easily accessible. In addition, the content of dietary fiber, vitamins, minerals and other grains is higher than that of most cereals, and it has higher nutritional value than traditional staple foods such as rice and wheat (1), which is called “golden cereal.” The United States introduced quinoa to NASA as a daily ration for astronauts. In addition, the FAO recognizes quinoa as the only food that can satisfy all the nutritional needs of human beings in a single crop and promotes and publicizes quinoa. The United Nations designated 2013 as the International Year of Quinoa to call attention to food security and balanced nutrition. China’s quinoa large-scale (more than 10,000 acres) planting is mainly distributed in Gansu, Qinghai, Yunnan, Shanxi, Inner Mongolia, Hebei. Other provinces are mostly sporadic or experimental planting; quinoa products are processed for the family workshop, large-scale, modern processing enterprises are less. China’s quinoa planting area and total output have jumped into the world’s top three, and the planting area reached about 20,000 hectares, and the output reached 28,800 tons in 2019. Shanghai, as a cosmopolitan city with 24 million permanent residents, the income and consumption level of urban residents have gradually increased, and the concept of dietary nutrition and health is also improving in recent year. Quinoa products have gradually entered the Shanghai consumer market, and the public’s consumption of quinoa products with comprehensive and balanced nutrition has gradually increased. However, it should also be recognized that the level of trust in the nutritional attributes of quinoa among urban residents determines their willingness to pay and sustained purchasing behavior. In recent years, with the increase in disposable income and health awareness of Chinese residents, higher requirements have been put forward for the nutritional value of food (2). In urban areas, this is reflected in increased consumption of foods with high nutritional value by consumers (3). In order to meet the market demand and promote the high-quality development of the whole quinoa industry chain, this study aimed to analyze the impact of nutritional trust and subjective norms of consumers in urban areas on the purchase behavior of quinoa products from the consumer side.

It is worth noting that, on the one hand, it is difficult to change the long-established dietary habits of consumers based on wheat or rice as a staple food (4). On the other hand, in some areas, there is a social culture that considers the nutritious amaranth foods such as quinoa as “poor foods.” These are all significant factors that discourage consumers from purchasing quinoa products (5, 6). Consumers’ purchasing behavior for healthy foods such as buckwheat and soy products is affected by the appearance of the food (7), smell (8), taste (9), and cooking convenience (10, 11) and shelf-storage implications. When purchasing, consumers are more likely to buy products with high brand familiarity (12), attractive packaging (9), easy availability of products (13), and affordable prices (14, 15). As consumers become more educated (16, 17), their intrinsic nutritional value of food (Hernandez (18, 19)) and environmental sustainability (20) will be the driving force of their purchasing behavior. Considering the influence of social pressure, imperative subjective norms such as the advice of doctors or nutrition experts (21), the consumption habits of female family members (22), and commercial promotion can also influence individual purchasing decisions. Consumers’ willingness to purchase products is influenced by the price and packaging of the product (12), the environmental friendliness of the food growing process, the social responsibility of retailers (23), the familiarity of consumers with the product (24), and the demographic structure of the family (25).

The existing literature analyzes the influence of food nutritional value and prescriptive subjective norms on purchasing behavior. The impact on trust and descriptive subjective norms needs to be expanded. In terms of research methods, principal component analysis, cluster analysis, joint analysis, structural equation model had been used to empirically analyze the influencing factors of consumer purchasing behavior, but there is a lack of heterogeneity analysis of the survey samples. In view of this, based on 1,078 micro-survey data from 16 administrative regions of Shanghai in July 2021, this paper uses ordered logistic model to empirically test the influence of trust in nutrition and subjective norms on consumers’ purchase behavior and willingness to pay for quinoa products, and further examines the moderating effect of individual preference heterogeneity. This study is great significance for the development of the quinoa industry throughout China and even globally, as well as the promotion of the transformation and upgrading of urban residents’ consumption of nutritious and healthy agricultural products.

## 2 Theoretical foundation

### 2.1 Analysis of the influence of trust in nutrition on purchasing behavior and willingness to pay

The core connotation of trust is a positive psychological expectation of someone for others or things (26), in which the person to organization (27, 28), individual (29), brand and other different objects of trust (30) have profoundly affected their purchasing behavior and willingness to pay. In the field of marketing, trust is often understood as the psychological process of creating trust in a transacting partner. Consumer trust is a kind of emotional acquiescence and dependence of consumers on the trusted party in terms of emotional attitude, and in terms of behavioral drive, it is the degree of belief in the characteristics of the object (31). Different products launched on the market are linked to different information and knowledge, and when consumers are confronted with a large amount of information, trust can reduce their perception of the uncertainty of a certain product, then results the higher level of trust (32) has a more significant role in promoting their willingness to buy and behavior (33). This article defines nutritional trust as consumers’ subjective confirmation that quinoa products have high nutritional value. Quinoa is an exotic crop, and the current market for quinoa products is still in the development stage, and e-commerce channels are the mainstay in product retail. Domestic consumers lack understanding of its nutritional characteristics, in the process of product selection, when consumers are exposed to a large amount of information about product characteristics from interpersonal communication, network and other channels, trust becomes a shortcut for consumers to process information (34). As an important value attribute of food (35), nutrition is a key element influencing consumers’ motivation to buy (36). Meanwhile, one study showed that consumer trust positively influences organic food purchasing behavior (37). Therefore, when consumers trust the nutritional value of quinoa products (38), it helps to promote their purchasing behavior and increase their willingness to pay. For example, the more satisfied consumers are with eco-labeled forest products, the more pronounced is their intention to consume eco-labeled forest products (39). Another study showed that the perceived value of green has a significant impact on consumer attitudes (40). From this, the hypotheses were proposed as follows:

*H1a*: Nutritional trust has a positive impact on the purchase behavior of quinoa products.

*H1b*: Nutritional trust has a positive impact on the willingness to pay for quinoa products.

### 2.2 Analysis of the influence of subjective norms on purchasing behavior and willingness to pay

Subjective norms refer to the social pressures of individuals to behave in a certain way, which is the attitude of support or opposition to specific behaviors generated by individuals such as spouses, doctors, or the expectations of groups such as friends, family, colleagues, etc. (41). Subjective norms include prescriptive norms and descriptive norms (42). Prescriptive norms are social pressures felt by others based on their supporting or opposing attitudes toward their own actions, and descriptive norms are social pressures generated by individuals based on observing or inferring the behavior of others. And descriptive norms have a stronger effect on individual behavior than prescriptive norms on individual behavior (43). As representatives of healthy foods, descriptive norms and purchasing behaviors were statistically significant (44). Consumers’ attitudes influence their intentions to purchase organic food, and subjective norms in turn influence attitudes (45). Due to the continuous participation and mutual influence of society and individuals and groups, and the influence of social and cultural factors, Chinese consumers show prominent characteristics in subjective normative perception. For example, Chinese consumers are easily influenced by others and show a tendency to obey high social expectations in behavioral decision-making (46). As a result, Chinese consumers attach great importance to external opinions and social acceptance, and tend to adopt behaviors that are consistent with social norms when making decisions (47). Studies have shown that most Chinese consumers say they believe that the opinions of those around them are credible, so they take the opinions of friends, family, celebrities, or influencers seriously when making decisions, and they also want to be accepted and value the feelings of others (48). In addition, one study showed that consumer attitudes and subjective norms had a significant positive effect on purchase intentions (40). Therefore, subjective norms are a very important factor in explaining the willingness to consume in the Chinese context (49). This paper focuses on the effects of descriptive subjective norms on individual behavior. When relatives, colleagues, classmates or friends in the surrounding circle choose to buy quinoa products, it helps to enhance consumers’ familiarity with the products, reduce the perception of purchase risk, and produce a positive impact on individuals’ purchase behavior and willingness to pay. Therefore, the hypotheses were proposed as follows:

*H2a*: Subjective norms have a positive impact on the purchase behavior of quinoa products.

*H2b*: Subjective norms have a positive impact on the willingness to pay for quinoa products.

### 2.3 Analysis of the moderating effect of preference heterogeneity

Attitude refers to the assessment and response of relatively stable positive or negative emotions to things (50), and individual preference is an essential factor influencing food purchase behavior (51). Heterogeneity is often studied in conjunction with moderating effects (52). However, the degree of heterogeneity can affect the difficulty of drawing overall conclusions (53). According to the International Food Information Commission (ICIF), the health attributes of food are the third most important food attributes after taste and price in the actual purchase scenario, influencing individuals’ consumption decisions about food from the perceptual level. Studies have shown that consumers use their experience to estimate the product they choose, determine whether the product meets their health expectations, and then make a purchase or rejection decision (54). Because quinoa products are not accepted by everyone in terms of taste compared to staple foods such as refined rice and white noodles, some consumers are not willing to sacrifice taste although they pay attention to food nutrition (55). In addition, in terms of cognition, if consumers do not understand the various attributes of quinoa products, their purchasing behavior will be more influenced by subjective norms, and the awareness of the nutritional benefits of food can prompt them to change their consumption preferences (56). Therefore, in terms of nutritional preferences, if individuals pay more attention to the nutritional value of food, it helps to enhance the impact of nutritional trust on purchasing behavior and willingness to pay. In terms of cognitive preferences, someone’s perception of quinoa products can weaken the influence of subjective norms on purchase behavior and willingness to pay. The hypotheses were proposed as follows:

*H3a*: Nutritional preferences have a positive moderating effect on the effect of trust in nutrition on purchasing behavior.

*H3b*: Nutritional preferences have a positive moderating effect on the effect of trust in nutrition on willingness to pay.

*H4a*: Cognitive preference has a negative moderating effect on the influence of subjective norms on purchasing behavior.

*H4b*: Cognitive preference has a negative moderating effect in the influence of subjective norms on willingness to pay.

**Figure 1:**
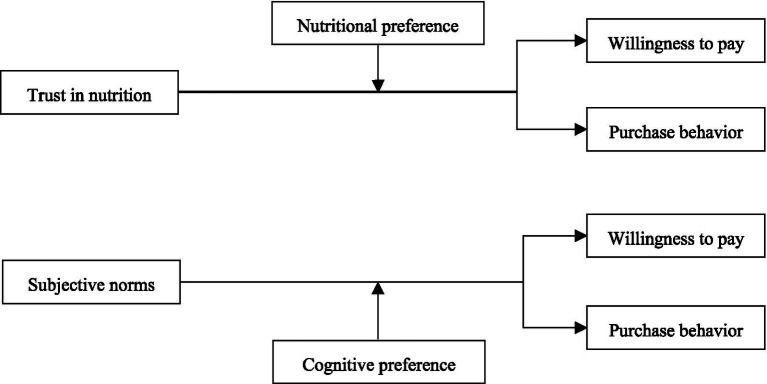
Analysis framework.

Figure 1 shows the analysis framework.

## 3 Research design

### 3.1 Empirical model

The consumer purchasing behavior of quinoa products is reflected by asking respondents whether they have purchased quinoa products before, with the options being “yes” or “no,” making it a typical binary variable (0–1 variable). The willingness to pay for quinoa products is reflected by asking respondents, “Considering the high nutritional value of quinoa and its very limited supply, what price per kilogram do you think is acceptable?” This includes five options: below 30 yuan/kg, 30–40 yuan/kg, 40–60 yuan/kg, 60–80 yuan/kg, and above 80 yuan/kg, making it a typical continuous variable with an ordinal relationship. Referring to previous books and literature (57–59), the binary logit model and the ordered logistic model were used to analyze the influencing factors of consumers’ purchase behavior and willingness to pay quinoa products, respectively, and the expression is shown as (Equations 1, 2):


(1) $$Y=\alpha_{0}+\alpha_{1}NT+\alpha_{2}SN+\alpha_{3}X_{i}+\varepsilon$$

where Y is the interpreted variable, indicating the purchase behavior and willingness to pay, NT and SN indicate trust in nutrition and subjective norms, Xi is the control variable group, α0, α1, α2, α3 are the regression coefficients, and the *ε* is a random error term.

In order to further test the moderating effect of nutritional preference and cognitive preference, the interaction terms of nutritional preference and trust in nutrition and the interaction terms of cognitive preferences and subjective norms are introduced, respectively, with the expression:


(2) $$\begin{array}{l} Y=\alpha_{0}+\alpha_{1}NT+\alpha_{2}SN+\alpha_{4}NUP+\alpha_{5}\mathrm{COP}\\ +\alpha_{6}NT\times NUP+\alpha_{7}SN\times \mathrm{COP}+\alpha_{3}X_{i}+\varepsilon \end{array}$$

where NUP and COP indicate nutritional preferences and cognitive preferences, respectively, and α4, α5, α6, and α7 are the parameters to be evaluated.

### 3.2 Data collection

**Figure 2:**
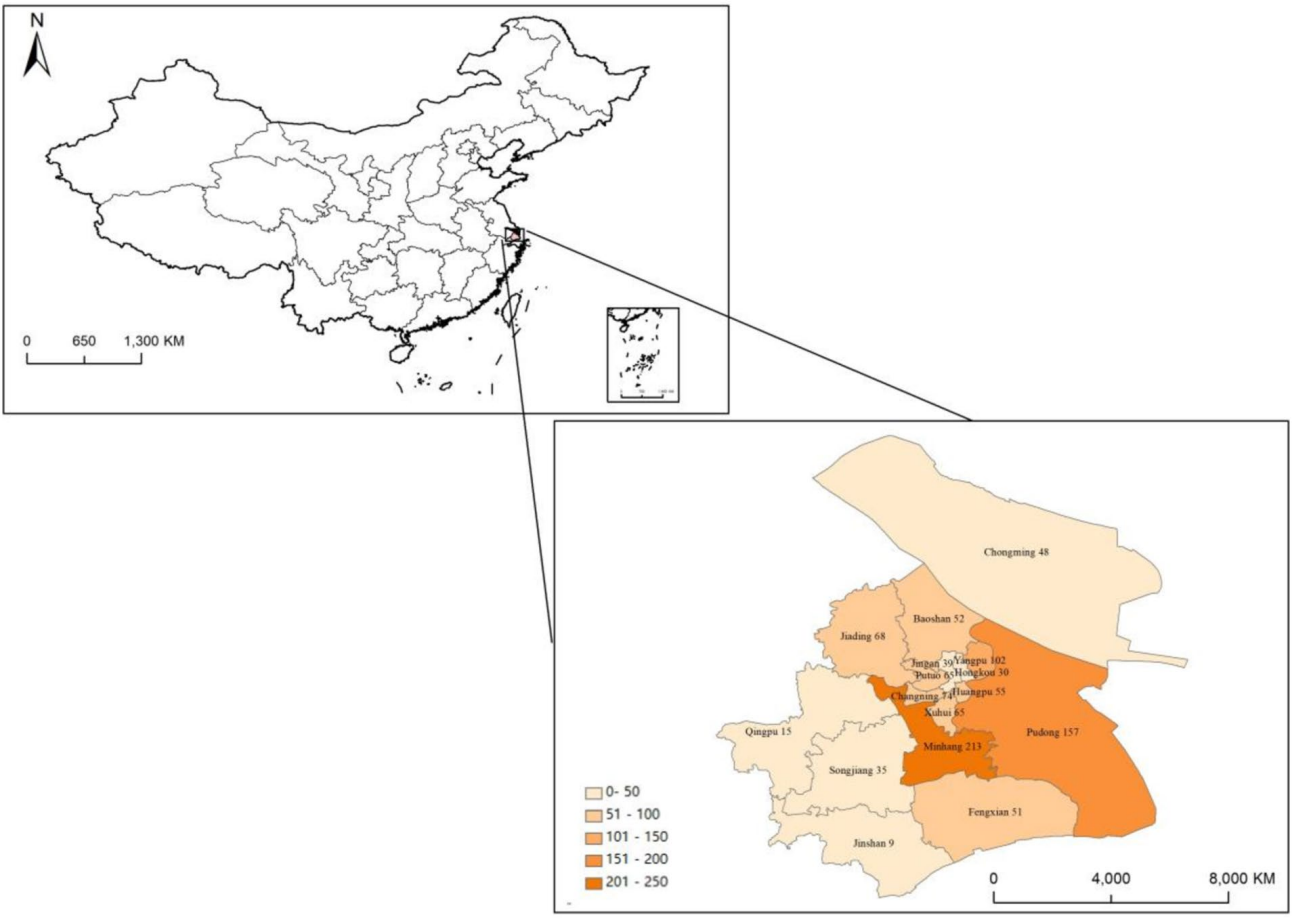
Sample size of each district.

The data in this paper comes from an online survey conducted by the research group on residents of 16 administrative districts in Shanghai through the “Wenjuanxing” online platform in July 2021, and 1,078 valid questionnaire data were finally obtained after excluding abnormal data samples. Wenjuanxing is a professional online survey platform in China, which allows users to conduct surveys through web pages or app mini-programs on both computers and mobile phones. The design of the survey mainly includes respondents’ basic personal information, daily consumption of agricultural products, as well as consumers’ awareness, attitudes, purchasing behavior, and willingness regarding quinoa products. The samples consisted of residents distributed in Minhang District (19.78%), Pudong New Area (14.56%), Yangpu District (9.46%), Changning District (6.86%), Jiading District (6.31%), Xuhui District (6.03%), Putuo District (6.03%), Huangpu District (5.1%), Baoshan District (4.82%), Fengxian District (4.73%), Chongming District (4.45%), Jing’an District (3.62%), Songjiang District (3.25%), Hongkou District (2.78%), Qingpu District (1.39%) and Jinshan District (0.83%). The sample size and proportion of each district are shown in Figure 2.

### 3.3 Sample characteristics

Based on the basic characteristics of the sample, the respondents are mainly under the age of 60, accounting for 94.16%, which may be related to the online survey method of the questionnaire. In terms of age distribution, residents aged 30 to 39 accounted for the most significant proportion, accounting for 33.77% of the total sample. Respondents were mainly registered residents of Shanghai, accounting for 66.98%. Overall, the respondents have a higher level of education, with 73.66% of respondents with bachelor’s degree or above. From the occupation perspective, civil servants, public institutions or enterprise employees accounted for 73.47% of the total sample. The average monthly household income of the respondents was concentrated at 30,000 Yuan or below, accounting for 60.20%. The respondents usually live with 1 or 2 children and elder, and the proportion of respondents with a family population of 5 or less is 80.15% (Table 1).

**Table 1:** Essential characteristics of sample.

Variable	Categories	Frequency	Proportion (%)
Gender	Female	667	61.87
	Male	411	38.13
Age	18 ~ 29	183	16.98
	30 ~ 39	364	33.77
	40 ~ 49	278	25.79
	50 ~ 59	190	17.63
	≥60	63	5.84
Census registration	Registered residents	722	66.98
	Non-registered residents	356	33.02
Education	Elementary school and below	3	0.28
	Junior high school	54	5.01
	Secondary school or high school	85	7.88
	Junior college	142	13.17
	Undergraduate	393	36.46
	Postgraduate and above	401	37.20
Occupation	Employees of government, institutions, or companies	972	73.47
	Respondents who work outside the government, institutions, or companies	286	26.53
Average monthly income (Yuan)	<10,000	215	19.94
	10,000 ~ 30,000	434	40.26
	30,000 ~ 50,000	108	10.02
	50,000 ~ 100,000	84	7.79
	100,000 ~ 150,000	62	5.75
	≥150,000	175	16.23
Number of family members	≤3	379	35.16
	4	205	19.02
	5	280	25.97
	6	205	19.02
	>6	9	0.83
Number of children in the family	1	550	51.02
	2	528	48.98
Number of elder in the family	1	511	47.40
	2	567	52.60

### 3.4 Definition and descriptive statistics

This study adopts consumers’ purchase behavior and willingness to pay for quinoa products as interpreted variables, while the core explanatory variables are trust in nutrition and subjective norms, and the moderating variables are nutritional preferences and cognitive preferences. Among them, trust in nutrition refers to consumers’ degree of trust in the nutritional comprehensiveness of quinoa products that exceeds that of any traditional food crop, which is a virtual variable. Subjective norms, nutritional preferences, and cognitive preferences are measured using five-point Likert scale method. In addition, in terms of controlling variables, five variables of respondents’ gender, age, census registration, education, and occupation were selected to measure personal characteristics. And family characteristics were measured by two variables: the number of elderly and children, average monthly income (Table 2).

**Table 2:** Variable definitions and descriptive statistics.

Variable type	Variable	Variable definition and assignment	Mean	Standard deviation
Interpreted variables	Purchase behavior	Have you ever purchased quinoa products? Yes = 1; No = 0	0.382	0.486
	Willingness to pay	Your acceptable price per kilogram of quinoa: under 30 yuan/kg = 1; 30 ~ 40 yuan/kg = 2; 40 ~ 60 yuan/kg = 3; 60 ~ 80 yuan/kg =4; more than 80 yuan/kg = 5	1.726	0.945
Core explanatory variables	Trust in nutrition	Do you believe that quinoa is more nutritiously comprehensive than any other traditional food crops? Believe very much = 1; Very unconvinced, not very convinced, or unsure = 0	0.271	0.445
	Subjective norms	Are there people in your surroundings who consume quinoa products? Few = 1; Rarely = 2; Passable = 3; A few = 4; A lot of = 5	1.941	0.840
Moderating variables	Nutritional preferences	If a food is nutritious but has a modest taste, would you rather consume it regularly? Very reluctant = 1; Not very willing = 2; Not necessarily = 3; More willing = 4; Very willing to = 5	3.115	0.979
	Cognitive preferences	How much do you know about quinoa? Never heard of =1; Only heard of it, not at all = 2; Passable = 3; More familiar, know that it has high nutritional value = 4; Very familiar with its high nutritional value and how to eat it =5	2.623	1.128
Controlling variables	Gender	Female = 0; Male = 1	0.381	0.486
	Age	18 ~ 29 = 1; 30 ~ 39 = 2; 40 ~ 49 = 3; 50 ~ 59 = 4; ≥60 = 5	2.616	1.132
	Census registration	Registered residents = 1; Non-registered residents = 0	0.670	0.471
	Education	Elementary school and below = 1; Junior high school = 2; Secondary school or high school = 3; Junior college = 4; Undergraduate = 5; Postgraduate and above = 6	4.921	1.144
	Occupation	Employees of government, institutions, or companies = 1; Respondents who work outside the government, institutions, or companies = 0	0.735	0.442
	The number of elderly and children	The sum of the number of elderly people over the age of 60 and the number of children under the age of 15 in the family	3.016	0.832
	Average monthly income	Average monthly household income after tax: ≤10,000 Yuan =1; 10,000 ~ 30,000 Yuan = 2; 30,000 ~ 50,000 Yuan = 3; 50,000 ~ 100,000 Yuan = 4; 100,000 ~ 150,000 Yuan = 5; ≥ 150,000 Yuan = 6	2.878	1.720

## 4 Results and discussion

### 4.1 Statistical analysis of the purchasing behavior of quinoa products

Judging from the purchase status of quinoa products of the respondents, 412 of the 1,078 respondents have purchased quinoa products, accounting for 38.22%, of which only 16.02% are purchased regularly. Most of the respondents considered the nutritional value of quinoa products to buy or just taste new food, and the purchase channels were mainly e-commerce platforms and supermarkets, accounting for 55.83 and 52.91%, respectively. Most of the quinoa products purchased by the respondents are produced in northwest China such as Gansu, Xinjiang and Inner Mongolia, but 40.53% of the respondents still do not know the origin of the products. Consumers mainly purchase raw quinoa, followed by adult nutritious foods such as quinoa cereals, lipid-lowering and sugar-lowering biscuits, and children’s nutritious foods such as quinoa flour. In terms of cooking, it is mainly mixed with other grains to make porridge or steamed alone. The taste, price, nutrition and freshness of the product are the main factors affecting the shopping experience, and only 33.25% of the respondents believe that the price of the product is more reasonable. Overall, more than half of the respondents had a better shopping experience. Among the groups that have purchased quinoa products, 74.27% of the respondents are willing to continue to buy quinoa products, and 80.34% are willing to recommend others to buy (Table 3).

**Table 3:** Overview of quinoa product consumption.

Variables	Categories	Frequency	Proportion (%)
Purchase behavior	Purchased	412	38.22
	Not purchased	666	61.78
Purchase frequency	Rarely purchased	98	23.79
	Occasionally purchased	248	60.19
	Regularly purchased	66	16.02
Reason for purchase	Comprehensive nutritional value	204	49.51
	Tasting new food	183	44.42
	Recommended by friends	97	23.54
	Fits the needs of family members	86	20.87
	Good taste	35	8.50
Purchase channels	E-commerce platform	230	55.83
	Supermarket	218	52.91
	Farmers market	50	12.14
	Others	26	6.31
	Roadside stall	8	1.94
Product form	Raw quinoa	295	71.60
	Nutritious foods for adults such as quinoa cereal, lipid-lowering and anti-diabetic biscuits	101	24.51
	Quinoa flour, biscuits, noodles, and other nutritious foods for children	92	22.33
	Drinks with quinoa	58	14.08
	Others	20	4.85
Product origin	Not clear	167	40.53
	Gansu, Xinjiang, Inner Mongolia and other northwest regions	132	32.04
	Yunnan and other southwest regions	54	13.11
	Other parts of China	30	7.28
	Overseas regions	29	7.04
Cooking method	Mixed with other grains	223	54.13
	Cook porridge or steam alone	112	27.18
	Eat with other kinds of foods	29	7.04
	Quinoa paste	25	6.07
	Quinoa tea	16	3.88
	Soup ingredient	7	1.70
Shopping experience	Very satisfied	37	8.98
	Relatively satisfied	185	44.90
	Passable	176	41.26
	Not very satisfied	7	1.70
	Very dissatisfied	7	1.70
The influencing factors of shopping experience	Taste	236	57.28
	Price	230	55.83
	Nutrition	168	40.78
	Freshness	160	38.83
	Safety	138	33.50
	Brand	70	16.99
	Production area	55	13.35
Price rationality	Very reasonable	8	1.94
	Comparatively reasonable	129	31.31
	Passable	212	51.46
	Not very reasonable	57	13.83
	Very unreasonable	6	1.46
Will you continue to buy quinoa products	Yes	306	74.27
	No	106	25.73
Will you recommend others to buy	Yes	331	80.34
	No	81	19.66

The reasons for purchase, purchase channels, product form, and the influencing factors of shopping experience are multiple choice questions.

### 4.2 Quantitative analysis of the influencing factors of the purchase behavior and willingness to pay for quinoa products

Before the empirical analysis, the variables are tested for multicollinearity, and the VIF value of each explanatory variable is up to 1.57, with the average value 1.21, which means that, there is no serious multicollinearity problem between the variables. In this section, the influencing factors of respondents’ purchase behavior and willingness to pay for quinoa products are analyzed by using binary logit model and ordered logistic model, respectively, and the LR chi-square test values are significant at the level of 1%, indicating that the results are statistically significant (Table 4).

**Table 4:** Binary logit model and ordered logistic model estimation results.

Variable	Purchase behavior		Willingness to pay	
	Coefficient	S.E.	Coefficient	S.E.
Trust in nutrition	1.202^***^	0.163	0.315^**^	0.140
Subjective norms	1.086^***^	0.103	0.381^***^	0.076
Gender	−0.500^***^	0.156	0.201	0.124
Age	0.149^*^	0.082	0.036	0.066
Census Registration	0.526^***^	0.186	0.382^***^	0.147
Education	0.013	0.073	0.047	0.059
Occupation	0.189	0.190	0.118	0.152
The number of elderly and children	−0.023	0.089	0.232^***^	0.072
Average monthly income	−0.007	0.044	0.038	0.035
Constant term	−3.699^***^	0.564	—	—
Observations	1,078		1,078	
LR chi2	307.33^***^		68.41^***^	
Pseudo *R*^2^	0.2143		0.0275	

***p < 0.01; **p < 0.05; *p < 0.1.

In terms of the influence on purchasing behavior, both trust in nutrition and subjective norms significantly positively influence purchasing behavior at 1% level. In terms of the impact on willingness to pay, nutritional trust and subjective norms significantly improve consumers’ willingness to pay at 5 and 1%, respectively. It shows that when consumers trust the nutritional value of quinoa products more than any other traditional crops, or are more easily influenced by others’ behavior, they tend to buy quinoa products and their willingness to pay is higher. In terms of consumers, on the one hand, the current consumer’s daily diet is gradually changing to the direction of eating health under the premise of eating enough and eating well, so more and more attention is paid to its nutritional value when purchasing food, and the trust in the nutritional value of food under this trend has become the driving force to promote their purchase behavior and improve their willingness to pay. On the other hand, when a certain type of product has not been fully marketed and consumers are unfamiliar with its characteristics. The behavior of the people around them has become an important reference for individual decision-making Therefore, it is more likely to produce herd behavior; if relatives, colleagues, and others in the individual particular social circle have purchased quinoa products, it will help the individual purchase decisions.

From the perspective of control variables, when consumers are registered residents in Shanghai, the positive impact on purchase behavior and willingness to pay is significant at the level of 1%. At the same time, significantly positively impacts the willingness to pay, and females. The number of elderly and children only has a significant positive effect on the willingness to pay. Female consumers and aged consumers are more inclined to buy quinoa products. This is because for multi-generational families, their household consumption decisions tend to pay more attention to the consumption needs of the elderly and children, and they are more willing to pay higher prices for the products needed by older people and children. Regarding purchasing behavior, the positive influence of female consumers on purchasing behavior may be caused by the fact that women are usually responsible for daily household consumption. In addition, 61.87% female sample proportion may also affect the estimated results (Table 1).

### 4.3 Analysis of the moderating effect of preference heterogeneity

In addition to trust in nutrition and subjective norms, understanding food nutrition preferences and characteristics constitutes the internal driving force of individual food consumption behavior, which is an essential factor affecting the purchase behavior and willingness to pay quinoa products. In this section, after the variable centralization of trust in nutrition, subjective norms, nutritional preferences and cognitive preferences, the interaction terms between dietary preferences and nutritional trust and the interaction terms between cognitive preferences and subjective norms are established respectively, and the moderating effect of nutritional preferences and cognitive preferences are examined (Table 5).

**Table 5:** Moderating effect test results.

Variable	Purchase behavior				Willingness to pay			
	Model 1		Model 2		Model 3		Model 4	
	Coefficient	S.E.	Coefficient	S.E.	Coefficient	S.E.	Coefficient	S.E.
Trust in nutrition	1.544^***^	0.158			0.392^***^	0.142		
Subjective norms			0.722^***^	0.123			0.345^***^	0.082
Nutritional preferences	0.180^**^	0.073			0.138^**^	0.064		
Nutritional preferences× Trust in nutrition	0.126	0.155			0.315^**^	0.137		
Cognitive preferences			1.575^***^	0.115			0.131^**^	0.062
Cognitive preferences× Subjective norms			−0.220^*^	0.125			0.053	0.062
Gender	−0.591^***^	0.146	−0.296	0.182	0.137	0.123	0.231^*^	0.125
Age	0.106	0.076	0.037	0.096	0.021	0.065	0.021	0.066
Census registration	0.491^***^	0.172	0.309	0.214	0.379^**^	0.147	0.366^**^	0.148
Education	0.100	0.069	−0.147^*^	0.084	0.078	0.059	0.023	0.059
Occupation	0.070	0.177	−0.031	0.221	0.095	0.151	0.099	0.153
The number of elderly and children	−0.080	0.084	−0.102	0.104	0.205^***^	0.072	0.230^***^	0.072
Average monthly income	0.021	0.041	0.037	0.052	0.050	0.035	0.044	0.035
Constant term	−1.295^***^	0.488	−0.047	0.604	—	—	—	—
Observation	1,078		1,078		1,078		1,078	
LR chi2	181.80^***^		558.84^***^		54.24^**^		68.74^***^	
Pseudo *R*^2^	0.1268		0.3897		0.0218		0.0276	

***p < 0.01; **p < 0.05; *p < 0.1.

After of introducing interaction terms, the core explanatory variables trust in nutrition and subjective norms still have a significant positive impact on purchase behavior and willingness to pay. And in terms of purchase behavior, the interaction items between cognitive preference and subjective norms are significantly negative at the level of 10%, which means cognitive preference has a negative moderating effect on the influence of subjective norms on purchase behavior. In terms of willingness to pay, the interaction term coefficient between nutritional preference and nutritional trust was significantly positive at the level of 5%, which means nutritional preference has a positive moderating effect on the influence of nutritional trust on willingness to pay. The main reason is that although consumers pay more attention to the nutritional qualities of food in the food purchase process, the taste of quinoa products is not very satisfactory, its high nutritional value is in line with the consumption needs of such people and this can enhance consumers’ willingness to pay. When consumers have a high degree of understanding of quinoa products, their purchase behavior relies more on subjective judgment based on the product information they know, and the role of individual subjective judgment will have a specific substitution effect on the herd purchase behavior caused by the influence of others behavior.

### 4.4 Robustness testing

In order to ensure the accuracy of the research conclusions, in this section, the substitution variable is used to replace the purchase behavior with the purchase frequency, and the ordered logistic model is used to test the moderating effect of preference heterogeneity, where the variable of the purchase frequency is assigned: no purchase = 0; few purchase = 1; occasional purchase = 2; frequent purchase = 3. Then, by changing the empirical method, the ordered probit model is used to analyze the moderating effect of preference heterogeneity on the willingness to pay (Table 6). The results show that the interaction between cognitive preference and subjective norms has a significant negative impact on purchasing behavior, while the interaction between nutritional preference and nutritional trust has a significant positive impact on payment willingness, both are significant at the level of 5%, which is basically consistent with the above empirical results, and the empirical results are stable.

**Table 6:** Robustness test results.

Variable	Purchase frequency				Willingness to pay			
	Model 5		Model 6		Model 7		Model 8	
	Coefficient	S.E.	Coefficient	S.E.	Coefficient	S.E.	Coefficient	S.E.
Trust in nutrition	1.577***	0.147			0.221***	0.083		
Subjective norms			0.837***	0.116			0.196***	0.048
Nutritional preferences	0.207***	0.071			0.070*	0.037		
Nutritional preferences× Trust in nutrition	0.201	0.143			0.178**	0.080		
Cognitive preferences			1.575***	0.096			0.078**	0.036
Cognitive preferences ×Subjective norms			−0.196**	0.093			0.031	0.036
Controlling variables	Controlled		Controlled		Controlled		Controlled	
Observations	1,078		1,078		1,078		1,078	
LR chi2	218.22***		663.25***		54.17***		71.58***	
Pseudo *R*^2^	0.0988		0.3003		0.0218		0.0284	

***p < 0.01; **p < 0.05; *p < 0.1.

### 4.5 Discussion

The contribution of this study is highlighted in the following two aspects: first, combing through the existing literature, no relevant studies on quinoa consumption behavior have been found. And no empirical studies were found on the influence of consumer trust in the nutritional attributes of quinoa on the consumption behavior of quinoa products; second, in this study, trust theory was applied to explain the influence and mechanism of the effect of nutritional trust in quinoa products on consumers’ quinoa purchasing behavior and willingness to pay. Of course, we also recognize that at the current stage, compared to ordinary grain products, China’s quinoa production is relatively limited, and the prices of quinoa products in the market remain high, making quinoa products more characteristic of mid-to-high-end agricultural products. Urban residents in Shanghai have a higher income and consumption level, as well as a strong concept of dietary nutrition and health, which leads to a higher proportion of quinoa product purchases compared to most other regions in China. Therefore, the conclusions and recommendations of this study are more adaptable and transferable in large cities. The consumption behavior and influencing factors of quinoa products in China’s small and medium-sized cities and rural areas require further in-depth investigation, which is also the content we will continue to focus on and research in the future.

## 5 Conclusions and implications

### 5.1 Conclusion

This paper uses 1,078 consumer micro-survey data in Shanghai in July 2021 to empirically analyze the impact of trust in nutrition and subjective norms on consumers’ purchase behavior and willingness to pay for quinoa products and further explore the moderating effect of preference heterogeneity. The conclusions of the study are as follows: First, trust in nutrition and subjective norms can both promote consumers’ purchasing behavior and increase their willingness to pay for quinoa products. This shows that regarding the purchase behavior or the willingness to pay for the product, the consumers’ subjective level of trust in the nutritional value of the quinoa product and the influence of the behavior of the people surrounding consumers are indispensable influencing factors. Second, both purchasing behavior and payment willingness are moderated by preference heterogeneity, and consumers’ cognitive preferences will weaken the positive impact of subjective norms on purchasing behavior, while nutritional preferences can strengthen the positive impact of trust in nutrition on payment willingness. And, the above research conclusions are still valid after robustness testing.

The difference in the effect of preference heterogeneity on purchase behavior and willingness to pay may be due to the fact that the purchase behavior as a kind of actual action conducted by consumers is more complex than the willingness to pay in real situations. Even if the consumer is willing to pay a higher price for the consideration of the nutritional value of food, because of the need to consider the type of product, cooking methods, and other factors when purchasing, at this time, the consumer’s understanding of quinoa products and the purchase behavior of others will provide information reference for individual purchase decisions. At the same time, the improvement of the consumer’s own cognitive level can replace the herd consumption behavior caused by the influence of other people’s behavior to a certain extent.

### 5.2 Implications

Based on the research conclusions of this paper, the following policy suggestions are proposed: First, strengthening research and development of products oriented market-oriented. Manufacturers shall fully tap the consumption needs of the elderly, children, students, and other groups through extensive market research. At meantime, manufacturers shall optimize production process, and improve taste and enrich product types while retaining the nutritional value of quinoa and second, certification and labeling schemes for nutrition and other properties of products should be implemented to enhance public awareness. The consumers’ trust in quinoa products shall be and improved by implementing qualification assessment of manufacturers and nutrient testing and product certifications conducted by authorities. Third, intensifying publicity and enhance product promotion to enhance consumers’ awareness of healthy diet. The multi-actor publicity model which is government-led, multi-channel media disseminated and community participated can be used to lead the trend of healthy consumption, and promote consumers to develop a balanced diet model with appropriate intake of cereals and a variety of different foods.

## Data Availability

The raw data supporting the conclusions of this article will be made available by the authors, without undue reservation.
